# pGlcNAc Nanofiber Treatment of Cutaneous Wounds Stimulate Increased Tensile Strength and Reduced Scarring via Activation of Akt1

**DOI:** 10.1371/journal.pone.0127876

**Published:** 2015-05-08

**Authors:** Haley Buff Lindner, Lloyd McPherson Felmly, Marina Demcheva, Arun Seth, Russell Norris, Amy D. Bradshaw, John Vournakis, Robin C. Muise-Helmericks

**Affiliations:** 1 Department Regenerative Medicine and Cell Biology, Medical University of South Carolina, Charleston, SC, 29425, United States of America; 2 Department of Medicine, Medical University of South Carolina, Charleston, SC, 29425, United States of America; 3 Marine Polymer Technologies, Inc., Danvers, MA, 01923, United States of America; 4 Sunnybrook Research Institute, University of Toronto, Toronto, Ontario, M5G 1G6, Canada; University Hospital Hamburg-Eppendorf, GERMANY

## Abstract

Treatment of cutaneous wounds with poly-N-acetyl-glucosamine containing nanofibers (pGlcNAc), a novel polysaccharide material derived from a marine diatom, results in increased wound closure, antibacterial activities and innate immune responses. We have shown that Akt1 plays a central role in the regulation of these activities. Here, we show that pGlcNAc treatment of cutaneous wounds results in a smaller scar that has increased tensile strength and elasticity. pGlcNAc treated wounds exhibit decreased collagen content, increased collagen organization and decreased myofibroblast content. A fibrin gel assay was used to assess the regulation of fibroblast alignment *in vitro*. In this assay, fibrin lattice is formed with two pins that provide focal points upon which the gel can exert force as the cells align from pole to pole. pGlcNAc stimulation of embedded fibroblasts results in cellular alignment as compared to untreated controls, by a process that is Akt1 dependent. We show that Akt1 is required *in vivo* for the pGlcNAc-induced increased tensile strength and elasticity. Taken together, our findings suggest that pGlcNAc nanofibers stimulate an Akt1 dependent pathway that results in the proper alignment of fibroblasts, decreased scarring, and increased tensile strength during cutaneous wound healing.

## Introduction

The inevitable result of adult wound healing is scar formation, which follows both surgical and trauma wounds. Scar formation following cutaneous wounding is due to an unorganized deposition of excess collagen and fibrous tissue that replaces normal dermal tissue. Scarring is a major medical complication that often results in restricted tissue movement, loss of function, poor aesthetics, and potential psychological distress [1]. The ability of mammals to heal without a scarring response (tissue regeneration), is restricted to a certain stage of development, after which wound healing proceeds with scarring. One major difference between tissue regeneration and scar formation is the organization and deposition of collagen. Comparisons of fetal regenerated tissue and adult scars show that each contains strikingly similar components, suggesting differences in regulation of tissue architecture and not in an abnormal molecular composition of the scar. Therefore tissue regeneration and scar-forming tissue repair share common mechanisms that may be controlled differently [2]. Fetal wound healing exhibit differences in the inflammatory response, TGFβ signaling, and hyaluronan synthesis. Fetal wounds are rich in hyaluronic acid which serves to facilitate an environment for cell migration and tissue regeneration. In adult wounds hyaluronic acid rapidly decreases while in fetal wounds it persists after persists for several weeks [3]. In addition, scarless wounds are characterized by a reduction in overall inflammatory responses that may be partly attributed to decreased platelet degranulation [4]. Decreased platelet activity results in reduced levels of TGFβ-1 and TGFβ-2 [4], which have established roles in the regulation of scarring. Reductions in these cytokines in fetal wounds correlate to reductions in scarring and addition of the cytokine to scarless wounds results in scar formation [5]. Interestingly, addition of TGFβ-3 to adult wounds leads to a decrease in scar formation suggesting the ratio of the differing isoforms is critical to tissue regeneration and scar formation [6]. Given the impact of scar formation and the limited effectiveness of current treatments such as silicone dressings and hydrocortisone injections, the development and characterization of new treatments are needed [7].

Our recent findings show that treatment of cutaneous wounds with pGlcNAc nanofibers (sNAG) an FDA approved material (TalyMed) promotes accelerated wound healing in both wild type animals and in those with delayed wound healing phenotypes in murine, pig, and human models [8–12]. Increased wound repair mediated by nanofiber treatment is characterized by increased hemostasis [13, 14]. Using the db/db mouse system, thin pGlcNAc nanofiber containing membranes placed directly into the wound bed profoundly accelerated wound closure mainly by re-epithelialization and increased keratinocyte migration, granulation tissue formation, cell proliferation, and vascularization compared with control wound treatments.

Mechanistic studies using these nanofibers *in vitro* and *in vivo*, have shown that these fibers directly bind to and stimulate integrin mediated outside/in signal transduction that is, at least in part, dependent on Akt1 activation [15, 16]. This activity appears to be due to their unique three dimensional beta quaternary structure. We have shown that nanofiber stimulation results in increased metabolism [17], increased endothelial cell motility and angiogenesis [16] and importantly to enhanced innate immune responses leading to antibacterial activity [8]. These findings indicate that pGlcNAc nanofibers may provide a combinatorial therapy using a single FDA approved agent.

Here we show that pGlcNAc nanofiber treatment results in reduced scarring where reduced scar size correlates with an increased tensile strength and elasticity of the healed wound. pGlcNAc treated wounds have a decreased collagen content, decreased myofibroblasts and increased collagen organization. We show that the serine threonine kinase Akt1 is required *in vivo* for the pGlcNAc-induced increases in tensile strength and elasticity. Using a fibrin gel assay to assess fibroblast alignment within a lattice that provides tension points, we show that nanofiber stimulation results in increased fibroblast alignment by a process also requiring Akt1. Our findings suggest that pGlcNAc nanofibers stimulate an Akt1 dependent pathway that results in the proper alignment of fibroblasts, decreased scarring, and increased tensile strength during cutaneous wound healing.

Increasing numbers of patients develop scars due to a rise in both elective operations and operations following traumatic injury [18]. While there are many current therapies on the market to reduce scar formation including flavonoid creams, silicone gel sheets, pressure therapy, corticosteroid injections, and cryotherapy, many of these therapies have resulted in controversial data and limited scientific studies [18]. Here, we evaluate a novel pGlcNAc nanofiber treatment that has been shown to increase wound healing while reducing bacterial infection. Our studies suggest that pGlcNAC treatment of cutaneous wounds results in decreased scar formation and improved tissue quality as measured by increased tensile strengths.

## Materials and Methods

### Ethics Statement

All experiments performed using mice were in accordance with animal procedure protocols approved by the Medical University of South Carolina Institutional Animal Care and Use Committee, approval ID# 2349. Appropriate analgesics were used under all conditions to insure a lack of pain and suffering.

### Excisional Wound Healing Model, Scar Quantification, Tensile Strength Measurements

Wild Type C57Bl/6 and Akt1 null mouse models [19] were used in all experiments. The Akt1 null animals were created using an insertional mutagenesis strategy at the translational start site that blocks expression of the entire protein [19]. Akt1 null animals on a mixed 129/C57Bl6 background were backcrossed onto C57Bl/6 for eight generations. Wounding was performed on anesthetized adult male mice between 8–12 weeks old. Two full thickness cutaneous wounds were created using a 4mm biopsy punch (Miltex), to create two identical wounds on each flank. Mice were anesthetized using an O2/Isoflurane vaporizing anesthesia machine (VetEquip, Inc.). Isoflurane was used at 4% for induction; 2% for surgery. Prior to surgery hair was removed by depilation and the area was washed and sterilized using 70% ethanol. Wounds were either treated with a thin dried form of pGlcNAc (sNAG membrane) obtained from Marine Polymer Technologies, Inc. which was moistened with distilled water or left untreated. On days of interest, animals were euthanized and entire wounds were harvested including the surrounding skin using an 8mm biopsy punch (Miltex). Wounds were fixed in 4% paraformaldehyde overnight at 4°C, embedded in paraffin, and sectioned for analysis.

For scar measurements (n = 6 for both untreated and treated controls), wild type mice were wounded as previously, or with a 1cm^2^ wound, which will not allow for contraction, and allowed to heal for 21 days. On day 21, animals were euthanized and scars were measured using a caliper.

For tensile strength, wounded animals were sacrificed after 21 days, wounds were harvested. Wounds, both treated and untreated and unwounded control skin was subjected to tensile strength and elasticity testing using an Instron 5942 strain gauge extensometer and Bluehill 3 Testing Software. Tensile strength of the skin was determined by measuring the relative stress the skin could bear before breaking 20% and elasticity was measured as the mm of extension. For tensile strengths, n = 6 for unwounded controls and for untreated, n = 5 for treated.

### Hematoxylin and Eosin Staining (H&E), Masson Trichrome Staining, Picro Sirius Red Staining

All H&E staining was performed in the Histology Core Facility at the Medical University of South Carolina, Department of Regenerative Medicine and Cell Biology. Briefly, sections were cleared in xylene, rehydrated through a series of graded alcohols, placed in hematoxylin followed by acid alcohol. Samples were then placed in ammonia water, rinsed in ethanol and exposed to Eosin before dehydrating through graded alcohols and clearing in xylene. Sections were mounted using Cytoseal-XYL (Richard-Allan Scientific). H&E sections were visualized using an Olympus BX40 microscope and captured using an Olympus Camera (Model DP25) and DP2-BSW acquisition software.

Masson’s Trichrome stain (Sigma-Aldrich) was performed according to manufacturer’s instructions for tissue sections. Briefly, sections were deparrafanized to water and incubated in Bouin’s solution. Slides were subjected to a series of incubations using hematoxylin, Biebrich Scarlet-Acid Fucshin, Phosphotungstic/Phosphomolybdic acid solution, Aniline Blue solution, and Acetic Acid as described by the manufacturer tissue sections were then dehydrated, cleared in xylene, and mounted using Cytoseal-XYL (Richard-Allan Scientific). Masson’s trichrome sections were visualized using an Olympus BX40 microscope and images were captured using an Olympus Camera (Model DP25) and DP2-BSW acquisition software.

### Extraction and Biochemical Quantitation of Collagen

Hydroxyproline assays were performed as previously described [20]. Briefly, equal amounts of wound tissue (8mm punch biopsy) was harvested from wild type mice treated or untreated at 21 days post wounding (n = 10 for untreated and n = 9 for treated). Tissue was lyophilized and weighed to ascertain dry weights and finely ground. Tissue collagen underwent complete acid hydrolysis with 6 N HCl for 18 h at 120°C, and neutralized to pH 7 with 4 N NaOH. One milliliter of chloramine T was added to 2-ml volumes of the collagen sample and incubated at room temperature for 20 min. One milliliter of Ehrlich's reagent (60% perchloric acid, 15 ml 1-propanol, and 3.75 g *p*-dimethyl-amino-benzaldehyde in 25 ml) was added, and samples were incubated at 60°C for 20 min. Absorbance at a wavelength of 558 was read on a spectrophotometer. Collagen was quantified as milligrams of hydroxyproline per gram dry weight of the wound tissue.

### Elastin Staining

Tissue sections from wounded animals, as described above, were stained for elastin fibers using Van Gieson staining procedures. Briefly, sections were cleared in xylene, rehydrated through a series of graded alcohols to distilled water, stained in hematoxylin (Sigma-Aldrich), differentiated in 2% ferric chloride and washed. Tissue sections were then stained in Van Gieson’s counterstain prior to dehydration, clearing in xylene, and mounting with Cytoseal-XYL (Richard-Allan Scientific). Tissue sections from at least n = 3 animals, treated and untreated were sections were visualized using an Olympus BX40 microscope and captured using an Olympus Camera (Model DP25) and DP2-BSW acquisition software.

### Immunofluoresence, microscopy

Paraffin embedded tissue sections were re-hydrated through xylene and a series of graded alcohols. Sections were treated with 0.01% Triton-X100 and subjected to antigen retrieval using antigen unmasking solution (Vector Laboratories) in a pressure cooker for 5 min and allowed to cool. Samples were incubated in Background Buster (Innovex Biosciences) for 30 minutes prior to antibody labeling. Skin sections were labeled with mouse monoclonal anti-Actin α-Smooth Muscle antibody (Sigma), Vimentin mouse monoclonal antibody (Dako), Phalloidin (Molecular Probes) and DAPI (Molecular Probes). Sections were incubated in primary antibody overnight at 4°C, washed, and incubated with the appropriate secondary immunofluorescent antibodies (Invitrogen) for 1 hour at room temperature. Control sections for each antibody were stained without primary antibody. Tissue sections were visualized using an Olympus FluroView laser scanning confocal microscope (Model IX70) and captured at ambient temperature using an Olympus camera (Model FV5-ZM) and Fluoview 5.0 acquisition software.

### Tissue Culture, Fibrin Gel Assay

C3H10 fibroblasts were maintained at 37°C with 5% CO2 in minimal essential medium with Earle’s balanced salt solution (MEM/EBSS, Thermo) supplemented with 1% penicillin/streptomycin (Invitrogen). Normal human fibroblasts (ATCC) were maintained in MEM supplemented with 20% FCS. Serum starvation was performed at 80–90% confluency in MEM/EBSS supplemented with 0.1% fetal calf serum (Valley Biomedical) for 24 hours followed by stimulation with highly purified pGlcNAc (50 μg/ml) nanofiber slurry (sNAG) in sterile water (provided by Marine Polymer Technologies, Inc.).

Fibrin Gel Assays were performed in 24 well tissue culture plates (Becton Dickinson). Plates were prepared for procedure by addition of 300 ml Sylgard 184 elastomer (Dow Corning) to the bottom of the well, which was allowed to set overnight at 37°C with 5% CO2. Two 0.1 mm minutien pins (Fine Science Tools) were inserted into the Sylgard along the (16mm) diameter of the well bottom, 3mm away from each edge. Wells were blocked with 3% BSA in PBS for 30 minutes. Cells were counted and resuspended in a fibrinogen solution composed of 5% human fibrinogen (Sigma) dissolved in cold MEM/EBSS media with the addition of 10μl/ml ascorbate and plated at a concentration of 250,000 cells per well. Thrombin (100units/mL) from human plasma (Sigma) was added to 5mg/mL fibrinogen and cell solution. The gels were incubated for 30 minutes at 37° with 5% CO2 prior to the addition of 1 ml of MEM/EBSS supplemented with 5% fetal calf serum. A small pipette tip was used to gently detach gels from the well walls. Gels were then monitored for contraction over time. At 6 to 8 hours after incubation, the media was removed, and the gels were washed 3 times with PBS, 5 minutes each and fixed with 4% paraformaldehyde for 30 minutes prior to either embedding in paraffin for tissue analysis or for phalloidin staining. For phalloidin staining: fibrin gels were treated with 0.3% Triton-X100 and incubated in phalloidin for 1 hour at room temperature, and counter-stained with DAPI for 10 minutes. Sections were mounted using fluorogel. For tissue analysis, fixed fibrin gel sections were subjected to H&E staining as described above.

### Lentiviral Infection

Mission shRNA lentiviral constructs directed against Akt1 were purchased from Sigma/Aldrich. Lentiviruses were propagated in 293T cells, maintained in DMEM supplemented as above. Lentiviral production was performed using psPAX2 and pMD2.G packaging vectors purchased from Addgene using the protocol for producing lentiviral particles from Addgene. For infection of target cells, 7.5 X 10^5^ cells were plated on 100 mm^2^ plates and allowed to incubate overnight. The next day, cells were transduced using a final concentration of 1mg/ml polybrene and Akt1 shRNA lentiviruses. After transduction, fibroblasts were serum starved overnight and stimulated with sNAG (50mg/ml). Any off target effects were controlled by verifying phenotypes using RNAi (Dharmacon) directed against a nonhomologous Akt1 sequence (data not shown).

## Results

### sNAG treatment results in decreased scar size and increased collagen alignment

**Fig 1:**
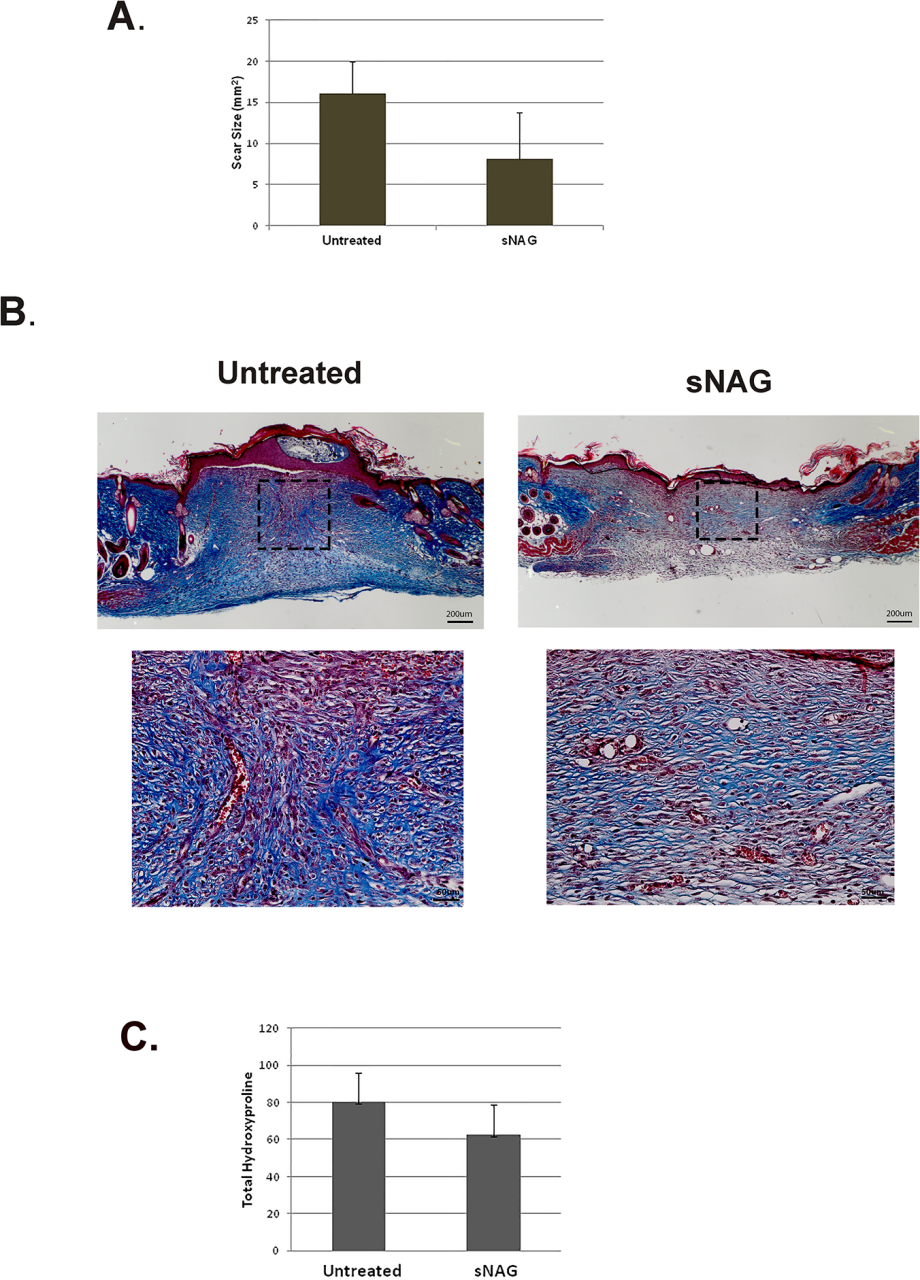
sNAG treatment results in decreased scar size and more organized collagen alignment. (**A**) Quantification of scar size from excisional wounds of Wild Type male mice (n = 6) that were either treated with sNAG membrane or left untreated. Scars were measured after 21 days of healing. (**B**) Masson Trichrome staining of paraffin embedded sections of cutaneous wounds harvested on day 10 post wounding from both WT untreated and sNAG treated mice. The 4x magnification illustrates the entire skin section while the 20x magnification is focused on the regenerating tissue directly under the wounded area. Boxes are included to show the regions of magnification. (**C**) Quantitative analysis of collagen content in sNAG treated wounds compared to untreated wounds of WT mice using a hydroxyproline assay, n = 10, untreated and n = 9 treated per group (p<0.05).

Our previous findings show that pGlcNAc nanofiber (referred herein as sNAG) treatment of cutaneous wounds results in increased wound closure in a diabetic mouse model [21] and in wild type mice [8] that is due, at least in part, to increased angiogenesis, keratinocyte proliferation and migration, and expression of several cytokines, growth factors [16] and innate immune activation [8]. We have noticed that at long time points post wounding it is very difficult to define the scar area. We therefore sought to analyze the effect of sNAG on scar formation. Excisional wounds were created in wild type animals which were either treated with a membrane form of sNAG or left untreated. At 21 days post-wounding, animals were sacrificed and scar sizes were measured. As shown by the quantitation in Fig 1A, sNAG treated wounds show an approximately 2-fold reduction in scar size as compared to untreated wounds. To examine the amount and quality of collagen in treated and untreated wounds, Masson’s trichrome staining was performed on tissue sections from 10 days post wounding. As seen in Fig 1B, sNAG treatment of cutaneous wounds results in decreased collagen content as indicated by less blue staining and more organized collagen alignment, especially as visualized at the wound borders, where new collagen in appropriately aligned with existing collagen. Hydroxyproline assays were performed to quantitatively analyze the amount of collagen deposition in treated and untreated wounds. As shown in Fig 1C, sNAG treated wounds have an approximately 3-fold decrease in overall collagen content.

**Fig 2:**
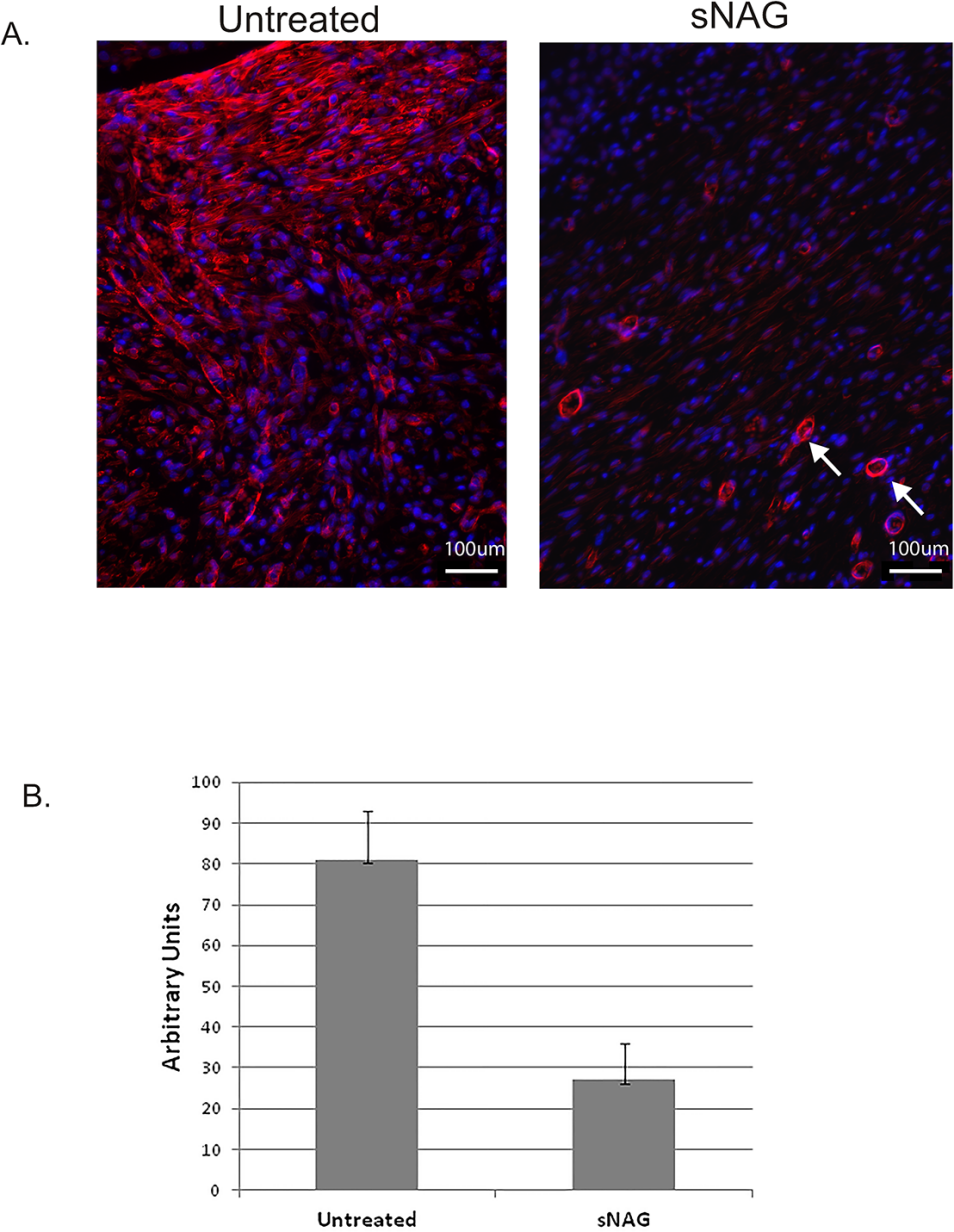
sNAG treatment results in decreased alpha smooth muscle expression. (**A**) Paraffin embedded sections of cutaneous wounds harvested on day 10 post wounding from both sNAG treated and untreated WT mice. Immunofluorescence (20X) was performed using antibodies directed against α-SMA (red), and TOPRO (Blue). White arrows indicate vasculature that is also positively stained with α-SMA antibodies. (**B**) Quantitation of α-SMA expression from paraffin embedded sections was performed using NIH ImageJ software. (*p<.05) n = 6 for each group.

Myofibroblasts are an important cell type in tissue repair and have been implicated in the generation of scarring via collagen production [22, 23]. Myofibroblast populations are reduced during fetal wound healing where scarring is absent [24]. To visualize the distribution of myofibroblast populations, wound sections were labeled with an antibody directed against alpha smooth muscle actin (αSMA). As shown in Fig 2A and quantitated in Fig 2B, treated wounds show at least a 2-fold reduction in the expression of αSMA, suggesting that the reduction in collagen content are due to reduced numbers of myofibroblast cell number.

### sNAG treatment causes increases in tensile strength and elasticity of wounded skin

**Fig 3:**
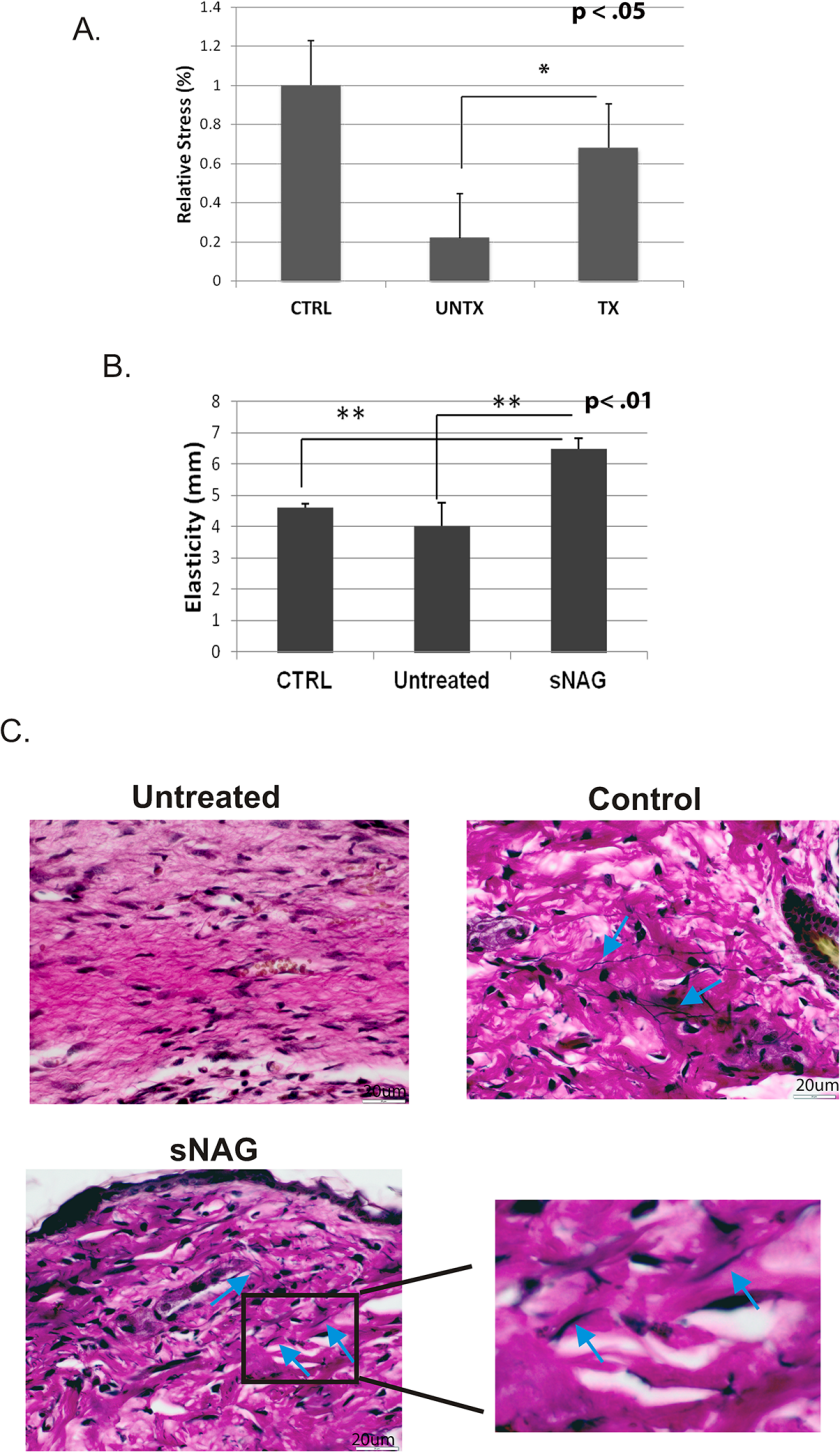
sNAG treatment causes increased tensile strength and elasticity of wounded skin. (**A**) Quantitation of the relative stress wounded skin could withstand from sNAG treated and untreated WT mice. Tissue was harvested 21 days post wounded and subjected to mechanical testing. Measurements are relative to control (CTRL) skin which was derived from unwounded tissue of WT animals. n = 6 for both unwounded CTRL skin and wounded untreated and n = 5 for treated wounds. (*p<.05) (**B**) Quantitation of skin elasticity from sNAG treated and untreated wounds harvested 21 days post wounding from WT animals. Control skin was derived from unwounded tissue of WT animals (**p<.01). (**C**) Van Gieson staining of paraffin embedded tissue sections derived from unwounded skin (control), sNAG treated, and untreated wounds of WT animals 10 days post wounding. Blue arrows indicate darkly stained elastin fibers.

Given that collagen imparts strength and mechanical properties [25] and that collagen deposition and scar formation differs between treated and untreated cutaneous wounds, we sought to measure whether sNAG treatment resulted in increased tensile strength and elasticity. To measure tensile strength and elasticity, cutaneous wounds generated in wild type animals, both untreated and treated, were harvested after 21 days and subjected to mechanical testing using a strain gauge extensometer. Normal unwounded skin from wild type mice was used as a control. Analysis of mechanical testing shows that sNAG treated cutaneous wounds of WT animals display an approximate 40% increase in tensile strength compared to untreated wounds (Fig 3A). Additionally, sNAG treated wounds exhibited tensile strength recovery at levels similar to unwounded control skin (Fig 3A). During tensile strength measurements, it was noted that sNAG treated cutaneous wounds from WT animals were more elastic than control or untreated counterparts. Fig 3B illustrates that sNAG treatment results in significantly increased elasticity of the healed tissue as compared to both untreated cutaneous wounds and unwounded control skin. To assess if sNAG treatment of cutaneous wounds results in increased elastin production, Van Gieson staining was analyzed. In Fig 3C, wounds derived from treated animals show elastin production (as visualized by the thin black structures) in the newly healed wound whereas untreated wounds do not. These findings suggest that sNAG treatment results in increased tensile strength as well as increased elasticity and supports the hypothesis that nanofiber treatment of cutaneous wounds decreases scarring while concurrently increasing mechanical properties of healing skin.

### sNAG treatment results in increased fibroblast alignment and contraction of fibrin gels

**Fig 4:**
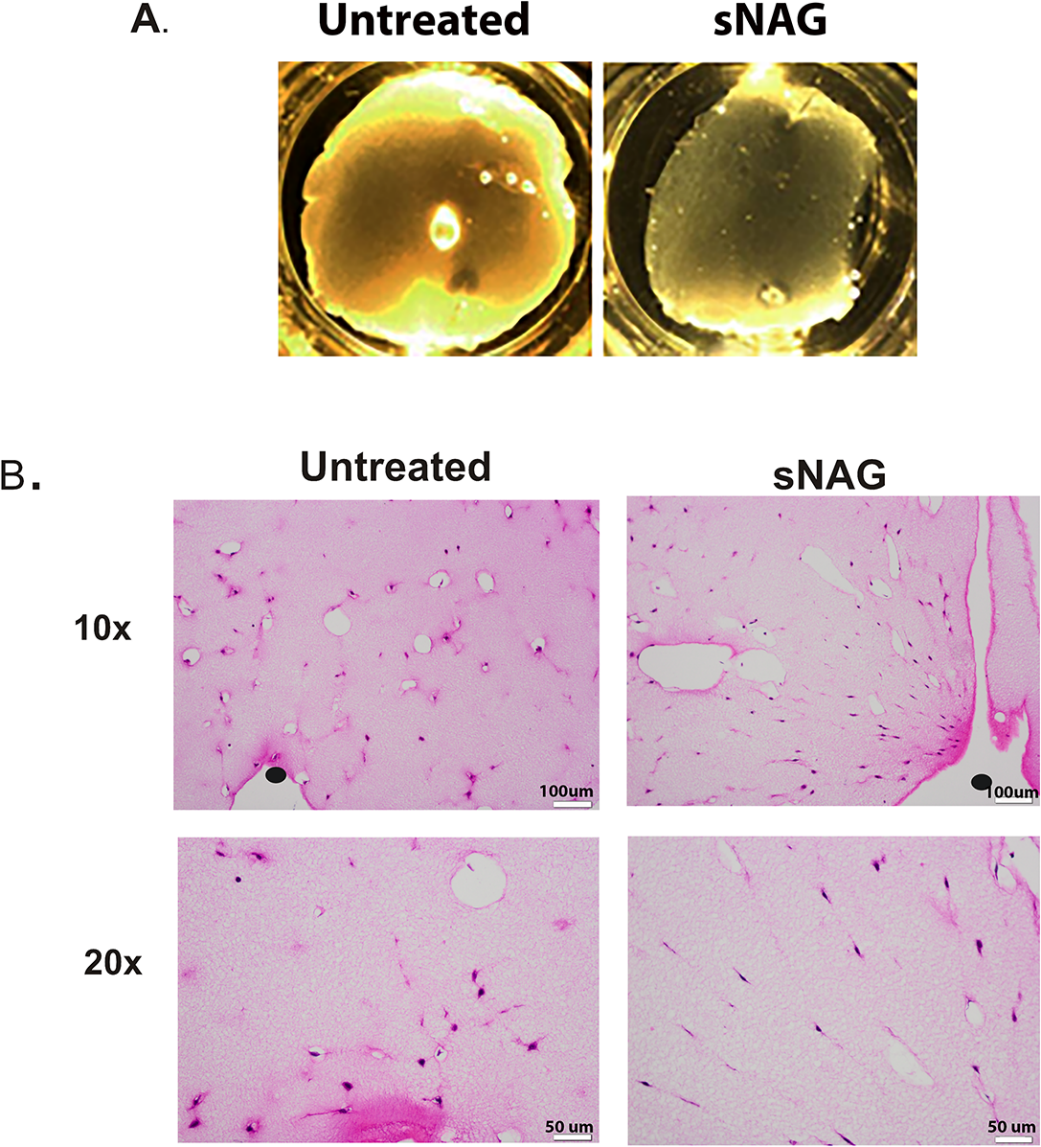
sNAG treatment results in increased fibroblast alignment. (**A**) Representative images of gel contractions. (**B**) Representative images of Hematoxylin and Eosin stained sections of paraffin embedded fibrin gels. Fibroblasts were serum starved and either left untreated or stimulated with sNAG (50μg/mL) overnight prior to embedding in fibrin gels. Black circles indicate where minutien pins are located in the fibrin gels to serve as tension points along which the gel contracts.

Since alignment and organization of collagen fibers is likely related to appropriate alignment of fibroblasts [26], we utilized a fibrin gel assay to assess if sNAG treatment results in alignment of fibroblasts *in vitro* [27]. To test this hypothesis, fibroblasts were treated with sNAG or left untreated prior to being embedded in fibrin gels. Fibrin gels were allowed to contract overnight and were then analyzed for contraction and sectioned for analysis of cell alignment. A representative photograph of sNAG treated and untreated fibrin gels is shown in Fig 4A. To visualize cell alignment, the gels were sectioned and stained with either phalloidin or H&E. Representative images of H&E stained gel sections show that sNAG treatment results in aligned fibroblasts (Fig 4B). Black circles indicate where pins were placed to allow tension points along which the gels could contract. sNAG-treated fibroblasts radiate from the pins in a linear, organized manner as compared to the scattered and unorganized arrangement of untreated cells. sNAG dependent fibroblast alignment was confirmed in human dermal fibroblasts (data not shown). In addition, sNAG-treated fibroblasts correlated with increased gel contraction as compared to untreated controls (see below). Taken together, these data suggest that sNAG treatment of fibroblasts results in organized cell alignment. This alignment of fibroblasts likely results in the more aligned collagen fibers and increased tensile strength shown in sNAG treated cutaneous wounds, as seen in Figs 1–3.

#### sNAG dependent fibroblast alignment requires Akt1

**Fig 5:**
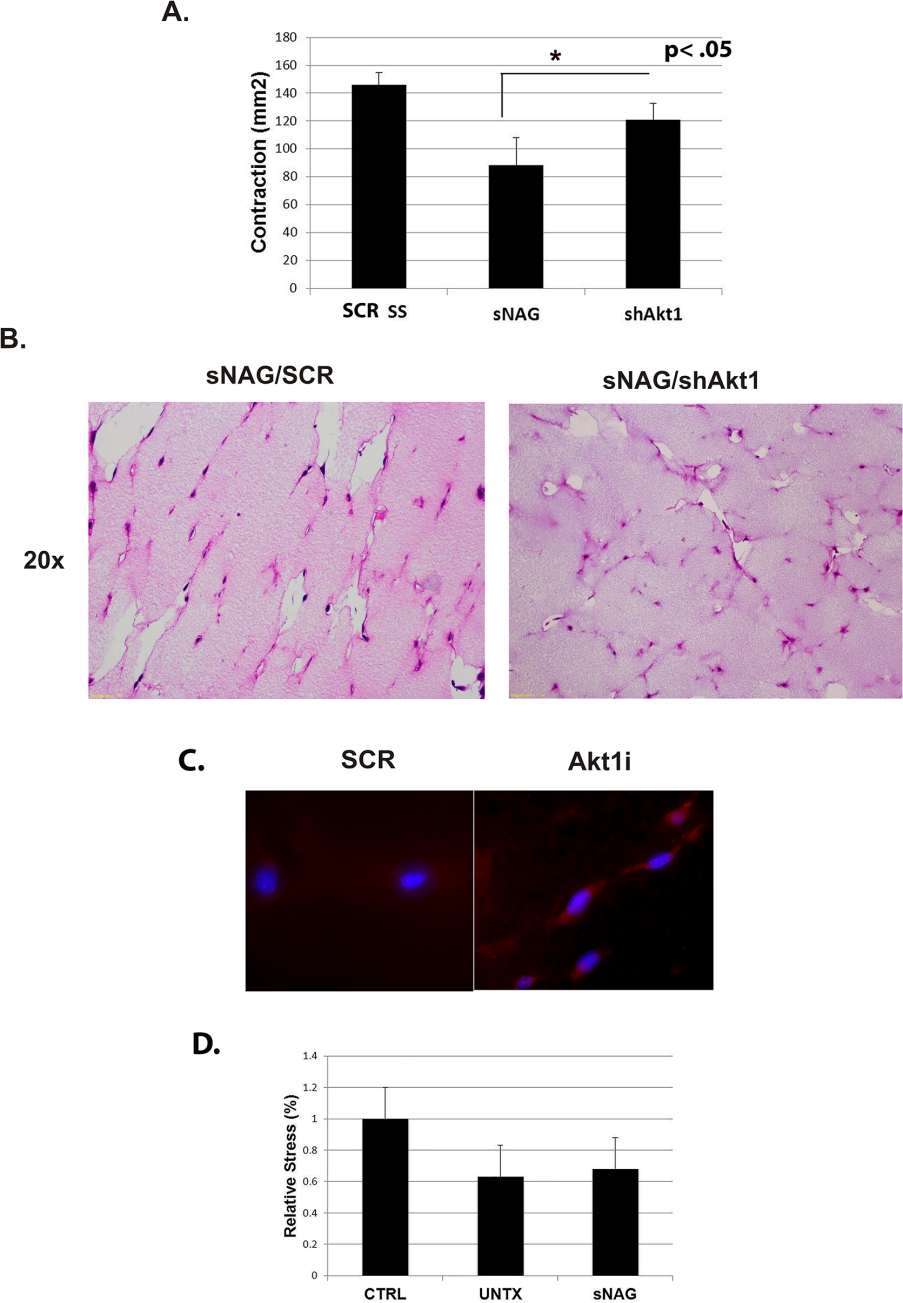
The sNAG dependent alignment of fibroblasts requires Akt1. (**A**) Quantification of contraction of fibrin gels that were embedded with serum starved fibroblasts that were transduced with a SCR control lentivirus, sNAG treated fibroblasts, or fibroblasts that were transduced with a lentivirus directed against Akt1 prior to sNAG stimulation (50μg/mL). (**B**) Representative images from Hematoxylin and Eosin stained sections of paraffin embedded fibrin gels. Fibroblasts were serum starved and transduced with either Scrambled control or Akt1 lentivirus prior to sNAG stimulation (50μg/mL). (**C**) Immunofluorescence of both serum starved (untreated) or sNAG treated fibrin embedded fibroblasts using an antibody directed against phospho-Akt (red) and DAPI. (**D**) Quantitation of the relative stress from sNAG treated and untreated Akt1 null animals n = 3 for both treated and untreated animals. Tissue was harvested 21 days post wounded and subjected to mechanical testing. Measurements are relative to control (CTRL) skin which was derived from unwounded tissue of Akt1-/- animals. (*p<.05)

Findings from our laboratory show that sNAG treatment results in increased cell motility and angiogenesis as a result of Akt1 activation [16]. Additionally, we have also shown that sNAG-dependent induction of innate immunity requires Akt1 [8] and that Akt1 null animals do not respond to sNAG treatment. To determine if Akt1 is also required for the sNAG-dependent increase in fibroblast alignment, fibroblasts were transduced with a shRNA expressing lentivirus directed against Akt1 prior to embedding in the fibrin gels. As shown in Fig 5A, sNAG-treated fibroblasts contracted the gels approximately 40% compared to gels containing untreated fibroblasts. Gels containing sNAG-treated shAkt1 expressing fibroblasts contracted significantly less than sNAG-treated controls. Analysis of H&E stained sections from the fibrin gels show that alignment is inhibited when Akt1 expression is blocked (Fig 5B). As shown in Fig 5C, sNAG-treated fibroblasts have an increased phosphorylation of Akt as compared to untreated controls, suggesting activation of the Akt pathway. Since the knockdown of Akt1 resulted in decreased fibroblasts alignment *in vitro*, we sought to determine if tensile strength of cutaneous wounds derived from Akt1 null animals would be affected by treatment with sNAG. Cutaneous wounds that had been treated with sNAG or left untreated were subjected to tensile strength testing using a strain gauge extensometer as described above. As shown in Fig 5D, there was no significant difference between sNAG treated and untreated wounds. Taken together, our findings suggest that Akt1 is required for the sNAG-dependent alignment of fibroblasts as well as sNAG-dependent increase in tensile strength *in vivo*.

## Discussion

Our findings show that highly purified pGlcNAc nanofiber (sNAG) treatment of cutaneous wounds results in reductions in scar formation, increased collagen organization in the absence of increased collagen, increased tensile strength and elasticity. These activities are blocked by Akt1 ablation both *in vitro* and *in vivo*. As tensile strength is determined by collagen remodeling during the maturation phase of wound healing we suggest that pGlcNAC nanofibers stimulate an Akt1 dependent pathway that leads to remodeling of collagen. Collagen remodeling is a complex process that involves both collagen synthesis and degradation by MMPs and other collagen binding proteins such as SPARC, followed by collagen crosslinking between the lysine and hydroxylysine of the peptides which is controlled by lysyl oxidase and lysyl hydroxylase which create stable bonds [28]. Akt1 is known to play a role in collagen synthesis through the control of the Ets family of transcription factors [29]. Ets family members are known regulators of MMP expression; e.g. MMP1 and 9. Taken together these findings suggest a central role for Akt1 in the regulation of scar formation. [30].

pGlcNAC nanofiber treatment leads to a more seamless collagen fiber organization, suggesting that sNAG treatment increases the kinetics of wound resolution and remodeling and decreases in α-smooth muscle actin (αSMA), suggesting a decreased number of myofibroblasts in the wound. These atypical, activated fibroblasts are the major producers of collagen in cutaneous wound healing, are active during contraction, and are cleared from the wound by apoptosis [23]. Our findings suggest that sNAG treatment effects the differentiation of myofibroblasts, their epithelial trans-differentiation, or their recruitment from the stroma or from the circulation. Since our model is a total Akt1 null animal, it is of interest to determine which cell type is responsible for the effects on myofibroblast recruitment or differentiation. Akt1 is known to stimulate cytokine secretion in both the endothelium [16] and in macrophage/monocyte lineages [31]. Myofibroblasts are thought to be recruited as circulating fibrocytes precursors. We therefore suggest that Akt1 may be controlling their recruitment.

To understand the role of sNAG in collagen alignment, we used a fibrin gel assay that provides tension lines on which cells can align and provides fibroblasts with a lattice similar to that which occurs during normal wound healing. Making the assumption that in order for collagen to be properly deposited, the fibroblasts themselves must be aligned. Cellular alignment is crucial for the formation of proper tissue architecture and provides appropriate mechanical function. Use of a three dimensional environment that more closely mimics an *in vivo* situation, provides an *in vitro* system in which to study these cellular responses. We show that pretreatment of fibroblasts with sNAG under serum starved conditions results in a marked alignment of cells toward the tension poles and that aligned cells physically contract the fibrin clot. Both the contraction and alignment is ablated under conditions of Akt1 knockdown. Interestingly, we see a slight but reproducible increased Akt phosphorylation in aligned cells. These findings show that Akt1 is required for sNAG-induced cellular alignment *in vitro*. Akt1 null animals validate these findings since the tensile strength of wounded skin is not affected by sNAG treatment in this model.

Exactly how sNAG activation of Akt1 results in fibroblast alignment remains to be determined. Akt signals to the cytoskeleton via the Rho-family of GTPases and PAC1, among others, controlling cytoskeletal remodeling [32]. Akt can directly phosphorylate vimentin, an intermediate filament protein involved in cell shape and motility changes [33]. In motile cells, phospho-Akt is localized to the leading edge whereas in apical/basal polarized cells, phospho-Akt tends to be localized to the cell periphery. These findings suggest that Akt plays a role in cell polarity, cytoskeletal organization and directed cell movement. The general consensus in fibroblasts, as determined by both knockdown and over expression analyses, is that Akt1 positively directs migration while Akt2 blocks migration [34]. The inverse is true in epithelial cells. In the fibrin gel assay presented here, Akt1 is required for the sNAG-induced alignment of cells between two tension points. Akt1 must then be required for cytoskeletal rearrangement and cellular positioning, possibly affecting front to back cell polarity. The exact mechanisms as to how Akt1 specifically affects this cell polarity in response to tension is currently under investigation. It is interesting to note that Akt1 null and wild type animals have similar tensile strengths of unwounded skin (data not shown). This suggests that the cellular programs controlling skin development are intact in the absence of Akt1. The double Akt1/Akt2 null animal, in addition to dwarfism and early neonatal lethality, presents with a thin skin phenotype, characterized by a marked reduction in the number of cells within each skin layer. This phenotype is due to a lack of proliferation that cannot be compensated by Akt3 alone [19]. Interestingly, the presence of either Akt2 or Akt3 does not affect sNAG stimulated wound repair and tensile strength as discussed here, or the clearance of infection and wound closure as previously reported [8]. This demonstrates that these two kinases are not functionally redundant for sNAG induced tissue repair or antimicrobial effects.

We also show that sNAG stimulation results in an increased elasticity as compared to both unwounded and wounded skin. Elasticity is conferred by elastin fibers formed during development. Elastin synthesis is absent in the adult but can be induced following injury through the up regulation of certain cytokines and growth factors, e.g. insulin-like growth factor-1 (IGFI) and interleukin 1 (IL-1), but is aberrantly deposited and is not detected until months after healing. Aberrant deposition of elastin leads to decreased elasticity and flexibility of the scar [35, 36] and is characterized by short, thin, immature fibers. sNAG treatment causes increased in elastin fiber staining that is visually similar to unwounded skin and correlates to increased trophoelastin protein expression and skin elasticity. Elastin expression is controlled, at least in part by IL-1 and IGF-1 and we show, at least *in vitro*, sNAG stimulation results in an increased expression of IL-1, suggesting a connection between this Akt1 and elastin fiber staining [16]. It is well established that Akt1 is a major kinase functioning downstream of both IL-1 and IGF-1.

In general, Akt1 has been linked to extracellular matrix assembly and fibronectin organization via the activation of β1 integrins [37]. Our previously published findings show that sNAG specifically activates integrin-dependent signaling and that this leads to Akt1 activation [15]. Therefore, it is possible that elastin deposition in the sNAG stimulated wounds is due to an integrin/Akt1 dependent pathway. Given that elastin fiber deposition does not generally occur in the adult, it will be important to understand the mechanisms by which sNAG stimulates elastogenesis for future application to skin repair and tissue bioengineering.

**Fig 6:**
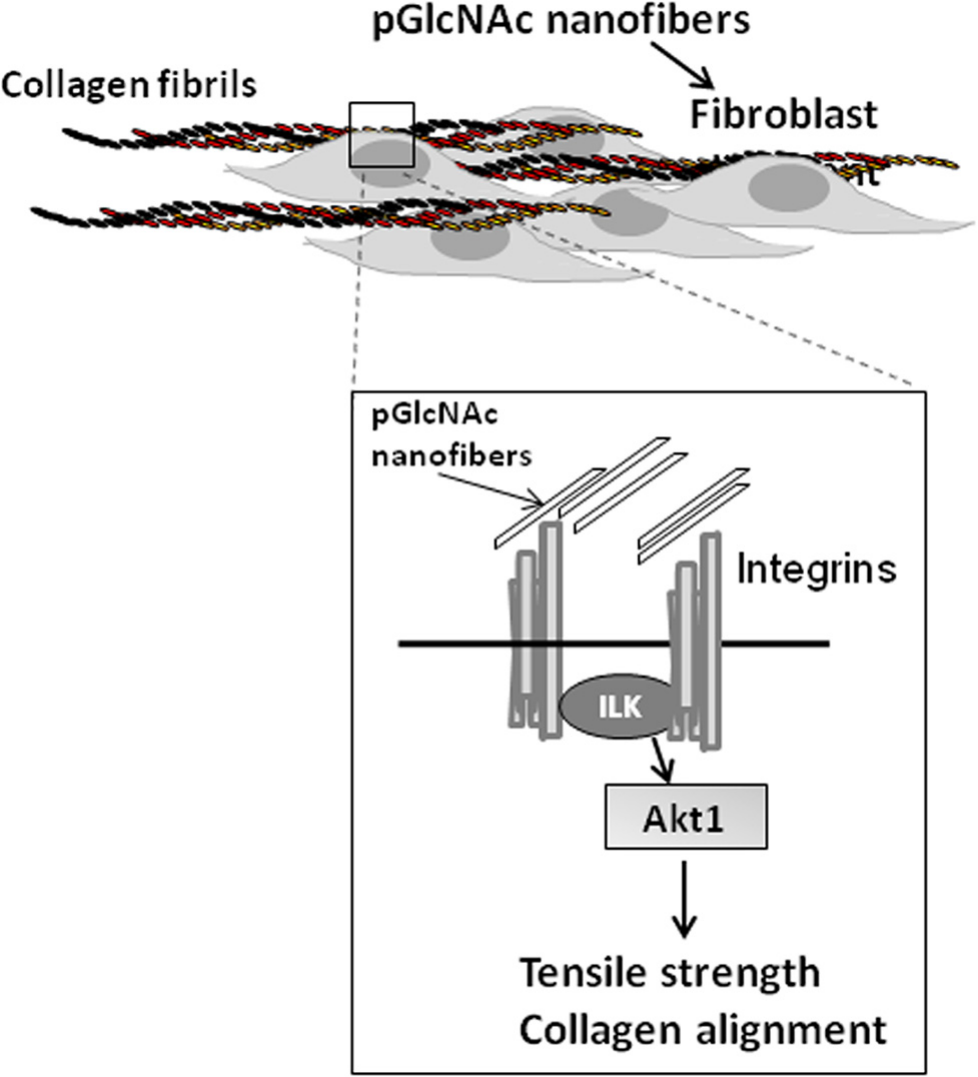
Model of sNAG-dependent regulation of collagen alignment and tensile strength. An illustration showing the interaction between pGlcNAC nanofibers and integrins, upstream of Akt1, that leads to increased collagen alignment and tensile strength in cutaneous wound healing.

Our findings suggest a model (Fig 6) by which sNAG nanofibers stimulate outside-in integrin signaling that results in an activation of Akt1 which controls cellular alignment under tension, and reductions in collagen I deposition and scar formation. Given that scarring is a major medical complication that causes decreased tissue function and aesthetics and the limitation of current treatment options, treatment of cutaneous wounds with this FDA approved material can be used to provide hemostasis, increased wound healing kinetics, increased innate anti-microbial responses and decreased scarring and increased function, thus providing combinatorial therapy using a single FDA approved agent.
